# Supplementary material for: The DEAD-box helicase eIF4A1/2 acts as RNA chaperone during mitotic exit enabling chromatin decondensation
Source: Nat Commun. 2025 Mar 11;16:2434. doi: 10.1038/s41467-025-57592-1 (PMC11897408; doi:10.1038/s41467-025-57592-1)
Supplement: Supplementary file 1 — Supplementary Information [file 41467_2025_57592_MOESM1_ESM.pdf]

Supplementary Information

The DEAD-box helicase eIF4A1/2 acts as RNA chaperone during mitotic exit enabling chromatin decondensation

Ramona Jühlen, Sabine C. Wiesmann, Anja Scheufen, Thilo Stausberg, Isabel Braun, Chantal Strobel, Carmen Llera-Brandt, Sabrina Rappold, Rabia Suluyayla, Marianna Tatarek-Nossol, Birgitt Lennartz, Hongqi Lue, Maximilian W. G. Schneider, Juan-Felipe Perez-Correa, Daniel Moreno-Andrés, Wolfram Antonin

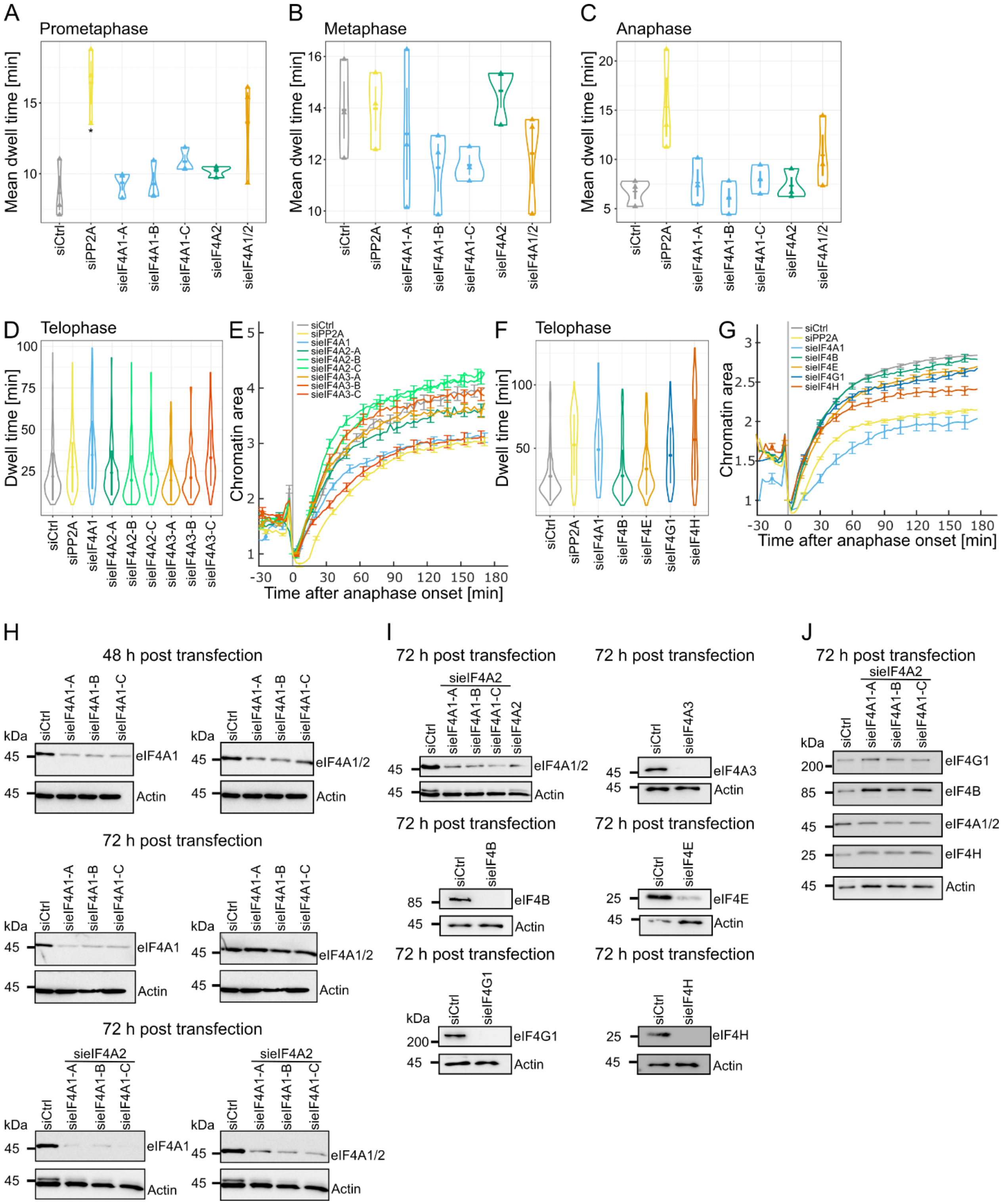

Supplementary Figure 1: Downregulation of eIF4F complex members (related to Fig. 2)

(A, B, C) Duration of early mitotic phases in HeLa cells stably expressing H2B-mCherry, transfected with 20 nM siRNA control or against PP2A, eIF4A1 (three different oligos), eIF4A2 or a combination of eIF4A1 (oligo A) and eIF4A2. Cells were analyzed by live cell imaging 48-63 h post-transfection. The mean time spent in

each mitotic phase is shown for at least 150 mitotic events per condition from three independent experiments. The violin plots show the means of three independent experiments (triangles) and the overall mean ± s.e.m.. Significance was tested by a two-tailed unpaired t-test with Welch's correction ((A) siPP2A, *P=0.02).

(D) Duration of telophase in HeLa cells stably expressing H2B-mCherry, transfected with 20 nM siRNA control or against PP2A, eIF4A1 (oligo B), eIF4A2 (three different oligos) or eIF4A3 (three different oligos). Cells were analyzed by live cell imaging 26-45 h post-transfection. The time spent in telophase is shown for more than 100 mitotic events per condition from one experiment. The violin plot shows the mean ± sd.

(E) Time-dependent quantitation of the chromatin area of the experiments presented in (D), normalized to metaphase-anaphase transition. Lines represent means ± s.e.m..

(F) Duration of telophase in HeLa cells stably expressing H2B-mCherry, transfected with 20 nM siRNA control or against PP2A, eIF4A1 (oligo A), eIF4B, eIF4E, eIF4G1 or eIF4H. Cells were analyzed by live cell imaging 26-72 h post-transfection. The time spent in telophase is shown for more than 30 mitotic events per condition from one experiment. The violin plot shows the mean ± sd.

(G) Time-dependent quantitation of the chromatin area of the experiments presented in (F), normalized to metaphase-anaphase transition. Lines represent means ± s.e.m..

(H) Western blot showing the downregulation of eIF4A1/2 at 48 and 72 h post-transfection with 20 nM siRNA oligos in HeLa cells stably expressing H2B-mCherry. In the left column, samples were analyzed with antibodies recognizing human eIF4A1, in the right column recognizing both human eIF4A1 and eIF4A2. Actin serves as loading control.

(I) Western blot showing the downregulation of eIF4F complex members at 72 h post-transfection with 20 nM siRNA oligos in HeLa cells stably expressing H2B-mCherry. Actin serves as loading control.

(J) Western blot showing the downregulation of eIF4A1/2 at 72 h post-transfection with 20 nM siRNA oligos in HeLa cells stably expressing H2B-mCherry. Actin serves as loading control. Example blots are representative for 3 experiments.

Source data are provided as a Source Data file.

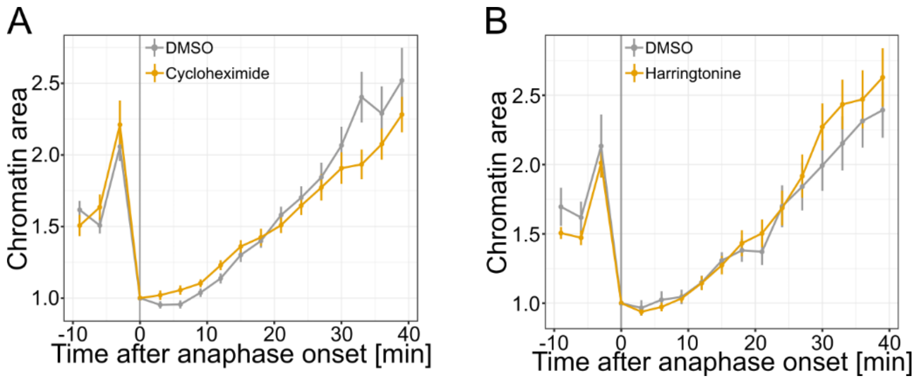

Supplementary Figure 2: Translation inhibition does not affect chromatin decondensation (related to Fig. 2)

(A) Time-dependent quantitation of the chromatin area of HeLa cells expressing H2B-mCherry after adding 50 µg/ml DMSO (control) or cycloheximide, normalized to the first anaphase frame. Dots represent mean ± s.e.m. from more than 15 cells per condition.

(B) Time-dependent quantitation of the chromatin area of HeLa cells expressing H2B-mCherry after adding 2 µg/ml DMSO (control) or harringtonine, normalized to the first anaphase frame. Dots represent mean ± s.e.m. from more than 10 cells per condition.

Source data are provided as a Source Data file.

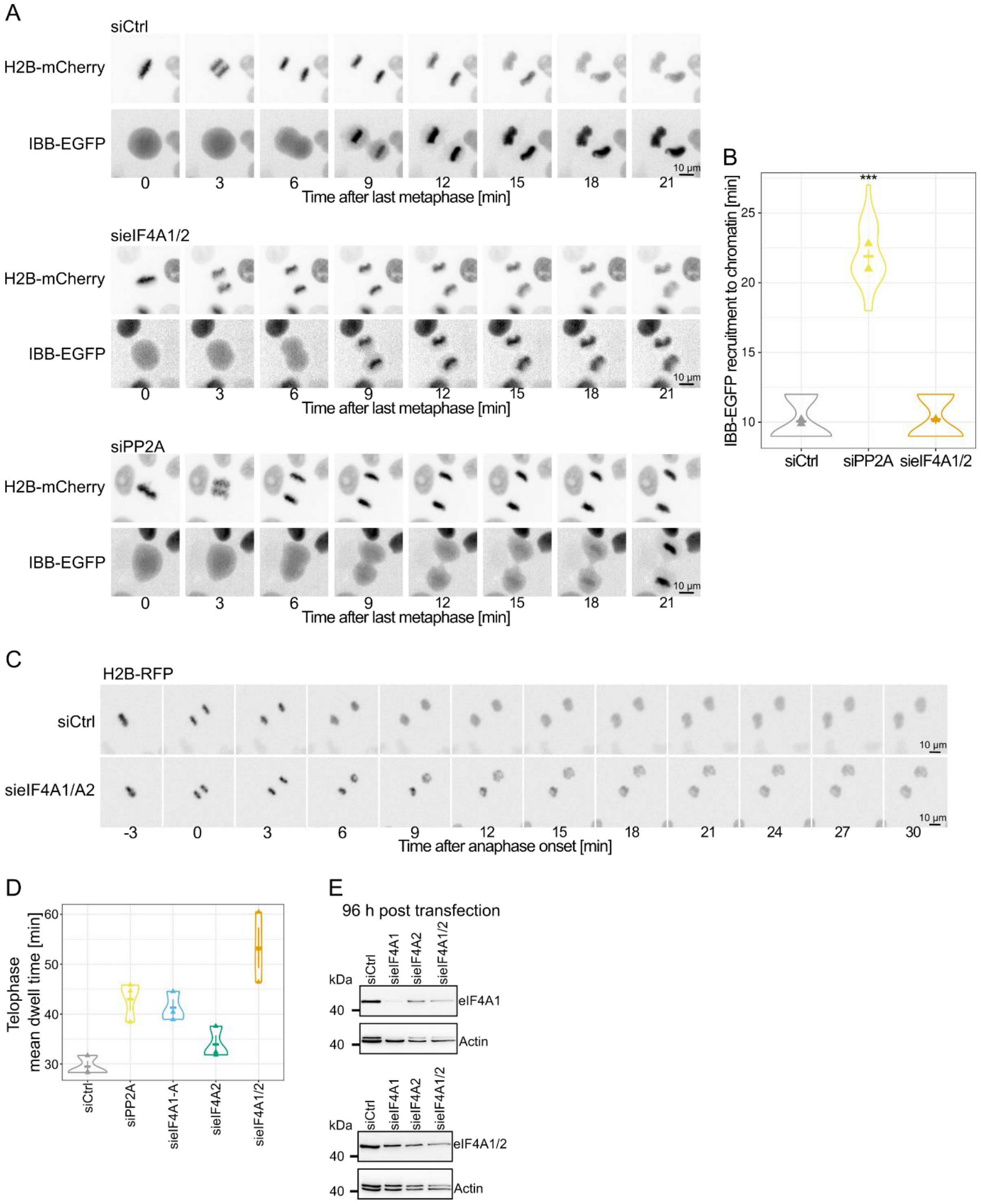

Supplementary Figure 3: HeLa IBB import/RPE cells: chromatin decondensation (related to Fig. 2)

(A) Time-lapse images of HeLa cells expressing H2B-mCherry and IBB-EGFP, transfected with 20 nM siRNA control, against PP2A or a combination of eIF4A1 (oligo A) and eIF4A2 for 48 h. Time is normalized to the last metaphase frame. Example images are representative for 2 experiments. Scale bars: 10 µm.

(B) Quantitation of the time of IBB-EGFP recruitment to the chromatin after the last metaphase frame in HeLa cells expressing H2B-mCherry and IBB-EGFP of the experiments as in (A). The violin plot shows the mean of two independent experiments (triangles) and the overall mean ± s.e.m. of in total 20 cells for each condition. At some points, errors might be too small to be visible. Significance was tested by a two-tailed unpaired Mann-Whitney U test (siPP2A, ***$P=2.46x10^{-8}$).

(C) Time-lapse images of RPE cells expressing H2B-RFP, transfected with 20 nM control or a combination of eIF4A1 (oligo A) and eIF4A2 siRNA oligos for 72 h. Time is normalized to the first anaphase frame. Example images are representative for 3 experiments. Scale bars: 10 µm.

(D) Telophase duration in RPE cells stably expressing H2B-RFP, transfected with 20 nM siRNA control, against PP2A or a combination of eIF4A1 (oligo A) and eIF4A2 siRNA oligos. Cells were analyzed by live cell imaging 48-72 h post-transfection. The mean time spent in telophase is shown for more than 70 mitotic events per condition from three experiments. The violin plot shows the means of three independent experiments (triangles) and the overall mean ± s.e.m..

(E) Western blot showing the downregulation of eIF4A1/2 at 96 h post-transfection with 20 nM siRNA oligos in RPE cells stably expressing H2B-RFP. In the upper left panel, samples were analyzed with antibodies recognizing human eIF4A1, in the lower panel recognizing both human eIF4A1 and eIF4A2. Actin serves as loading control. Example blots are representative for 3 experiments.

Source data are provided as a Source Data file.

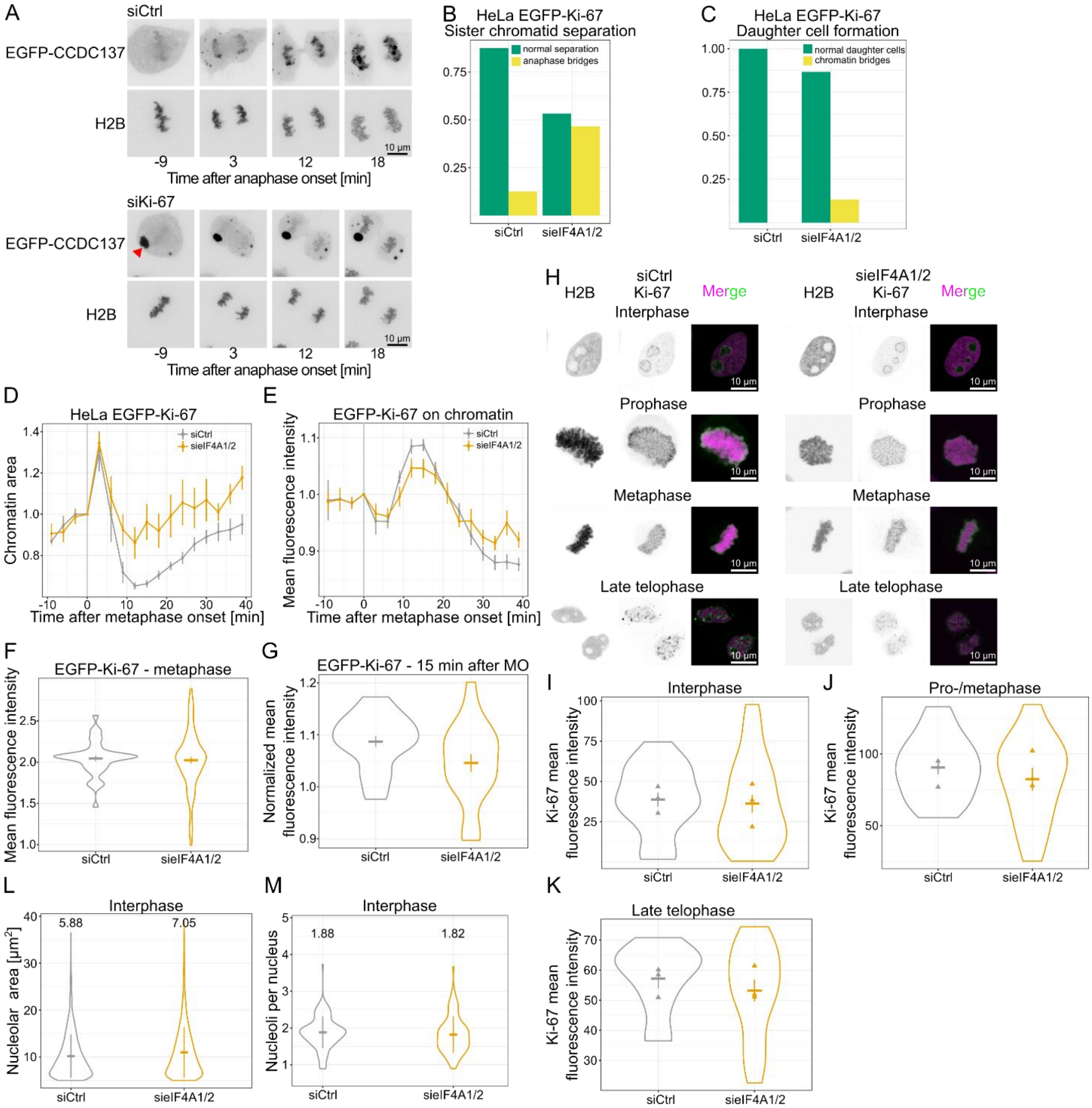

Supplementary Figure 4: Perichromosomal layer (related to Fig. 4)

(A) Time-lapse images of HeLa cells stably expressing H2B-mPlum and EGFP-CCDC137, transfected with 20 nM control or Ki-67 siRNA oligos for 72h showing cytosolic EGFP-CCDC137 aggregates (red arrowhead). Time is normalized to the first anaphase frame. Example images are representative for 3 experiments. Scale bars: 10 µm.

(B) Quantitation of failures during sister chromatid separation of the experiments as in (Fig. 4E) for more than 10 cells per condition.

(C) Quantitation of failures during daughter cell formation of the experiments as in (Fig. 4E) for more than 10 cells per condition.

(D) Time-dependent chromatin area analysis of the experiments as in (Fig. 4E), normalized to the last metaphase frame. Dots represent mean ± s.e.m. from more than 10 cells per condition.

(E) Time-dependent EGFP-Ki-67 mean fluorescence intensity analysis of the experiments presented in (Fig. 4E), normalized to the last metaphase frame. Dots represent mean ± s.e.m. from more than 10 cells per condition.

(F) Quantitation of the EGFP-Ki-67 mean fluorescence intensity as in (G) from metaphase cells presented as mean fluorescence intensity. The violin plot shows the mean ± s.e.m..

(G) Quantitation of the EGFP-Ki-67 mean fluorescence intensity as in (G) from cells 15 minutes after metaphase onset, normalized to the last metaphase. The violin plot shows the mean ± s.e.m..

(H) Ki-67 immunostaining of HeLa cells stably expressing H2B-mCherry, transfected with 20 nM control or a combination of eIF4A1 and eIF4A2 siRNA oligos for 72h, in interphase and at different stages of mitosis. Example images are representative for 3 experiments. Scale bars: 10 µm.

(I) Quantitation of the Ki-67 mean fluorescence intensity in interphase from three independent experiments as in (J). The violin plot shows the means of three independent experiments (triangles), each including more than 20 cells per condition and the overall mean ± s.e.m..

(J) Quantitation of the Ki-67 mean fluorescence intensity in pro-/metaphase from three independent experiments as in (J). The violin plot shows the means of three independent experiments (triangles), each including more than 10 cells per condition and the overall mean ± s.e.m..

(K) Quantitation of the Ki-67 mean fluorescence intensity in late telophase from three independent experiments as in (J). The violin plot shows the means of three independent experiments (triangles), each including more than 10 cells per condition and the overall mean ± s.e.m..

(L) Quantitation of the nucleolar area in fixed HeLa cells stably expressing H2B-mCherry, transfected with 20 nM control or a combination of eIF4A1 and eIF4A2 siRNA oligos for 72h. The violin plot shows the mean ± sd of at least 2000 cells. Mean values are given above each transfection.

(M) Quantitation of the number of nucleoli per nucleus in fixed HeLa cells stably expressing H2B-mCherry transfected with 20 nM control or a combination of eIF4A1 and eIF4A2 siRNA oligos for 72h. The violin plot shows the mean ± sd of more than 2000 cells. Mean values are given above each transfection.

Source data are provided as a Source Data file.

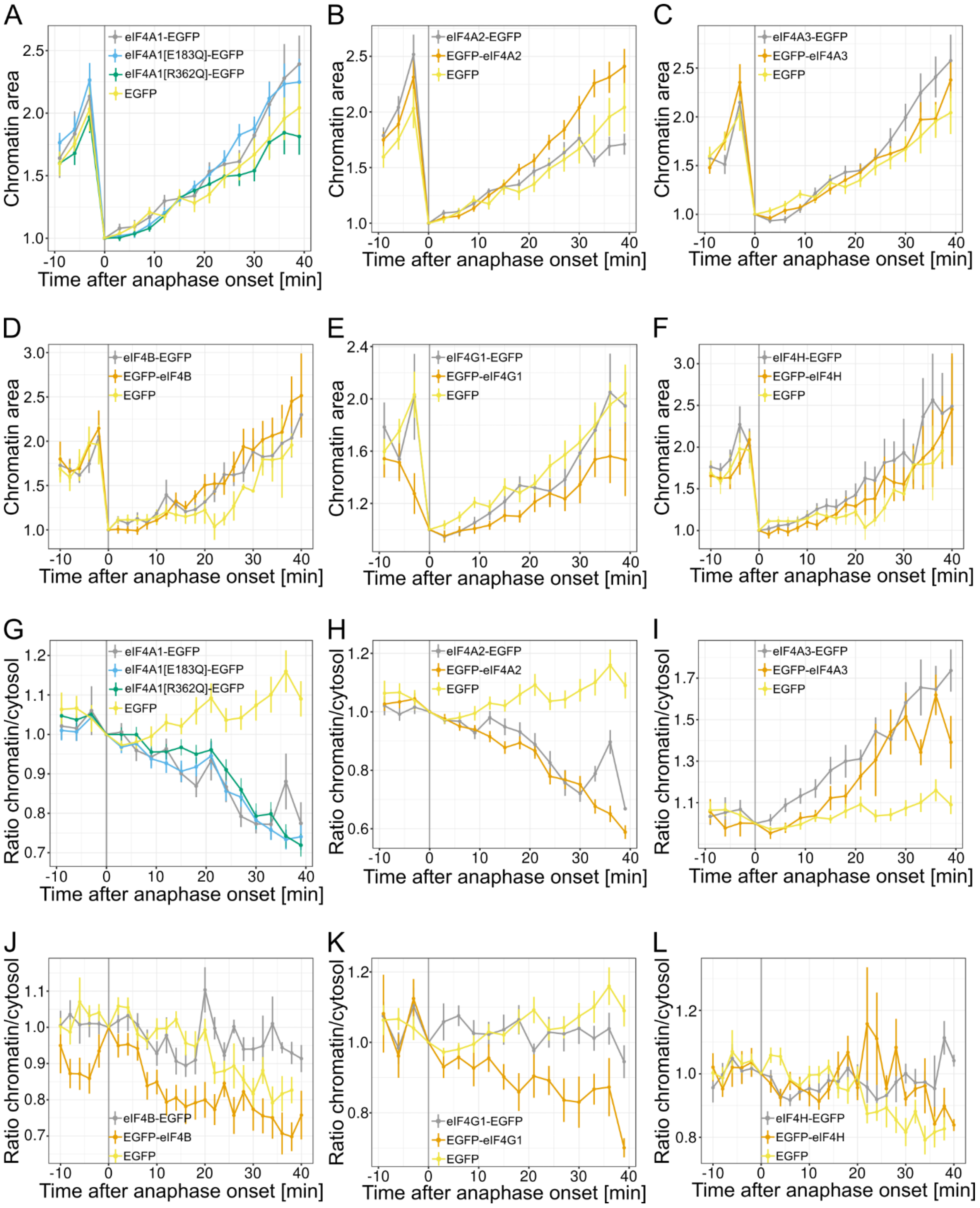

Supplementary Figure 5: Time-dependent quantification of chromatin area and fluorescence intensity of EGFP-tagged eIF4F complex members (related to Fig. 5)

(A) Time-dependent chromatin area analysis of the experiments with EGFP, eIF4A1-EGFP, the corresponding ATPase deficient mutant eIF4A1 E183Q or the RNA-binding deficient R362Q mutant presented in (Fig. 5E), normalized to the first anaphase frame. Dots represent mean ± s.e.m. from at least 10 cells per condition.

(B) Same as in (A) but with EGFP, eIF4A2-EGFP and EGFP-eIF4A2.

(C) Same as in (A) but with EGFP, eIF4A3-EGFP and EGFP-eIF4A3.

(D) Same as in (A) but with EGFP, eIF4B-EGFP and EGFP-eIF4B.

(E) Same as in (A) but with EGFP, eIF4G1-EGFP and EGFP-eIF4G1.

(F) Same as in (A) but with EGFP, eIF4H-EGFP and EGFP-eIF4H.

(G) Time-dependent mean fluorescence intensity analysis of the experiments with EGFP, eIF4A1-EGFP, the corresponding ATPase deficient mutant eIF4A1 E183Q or the RNA-binding deficient R362Q mutant presented in (Fig. 5E), normalized to the first anaphase frame. Dots represent mean ± s.e.m. from at least 10 cells per condition. The ratio of chromatin to cytosol is shown to compensate for different expression intensities of the cells.

(H) Same as in (G) but with EGFP, eIF4A2-EGFP and EGFP-eIF4A2.

(I) Same as in (G) but with EGFP, eIF4A3-EGFP and EGFP-eIF4A3.

(J) Same as in (G) but with EGFP, eIF4B-EGFP and EGFP-eIF4B.

(K) Same as in (G) but with EGFP, eIF4G1-EGFP and EGFP-eIF4G1.

(L) Same as in (G) but with EGFP, eIF4H-EGFP and EGFP-eIF4H.

Source data are provided as a Source Data file.

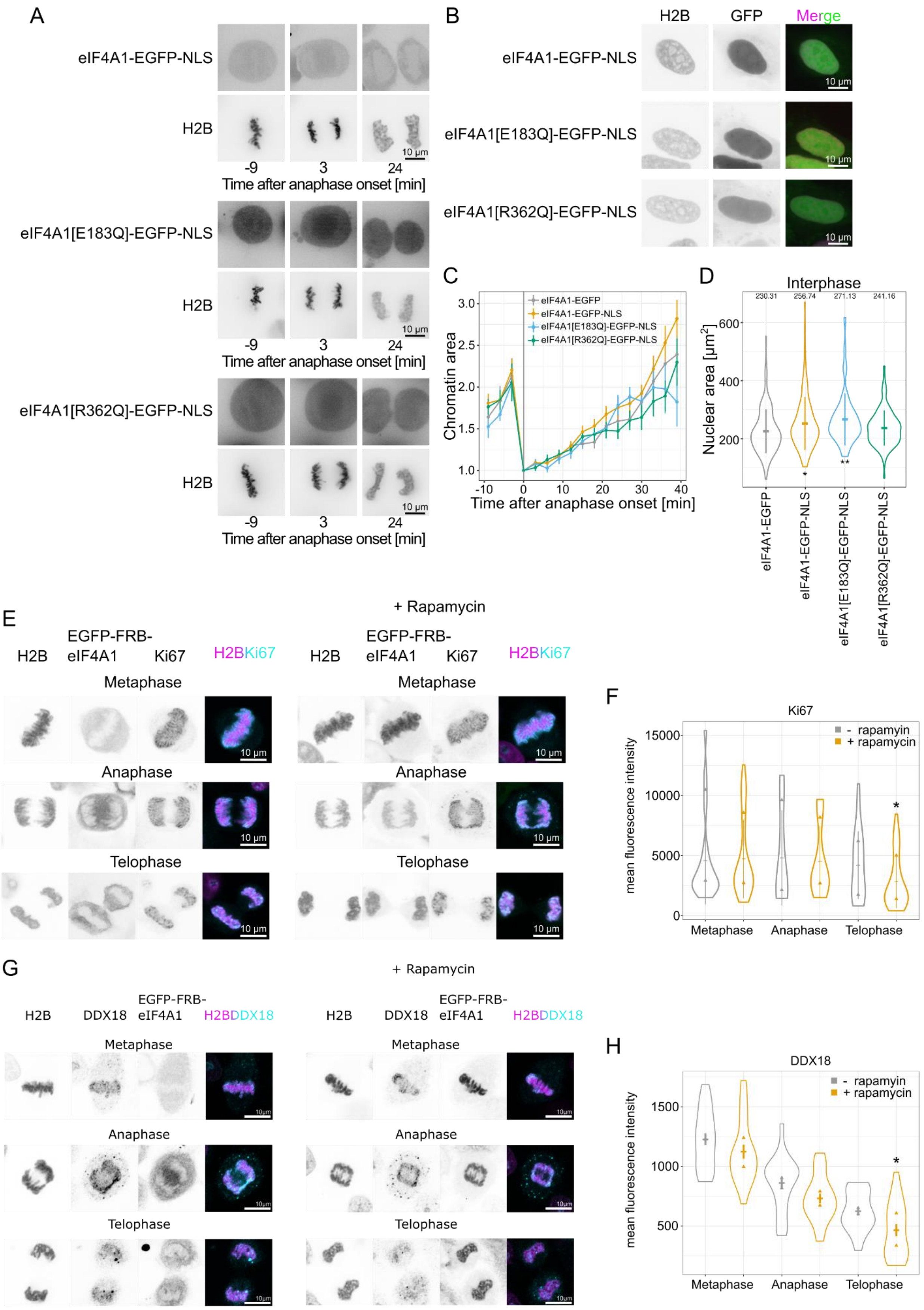

A
eIF4A1-EGFP-NLS
H2B
-9
3
24
Time after anaphase onset [min]
eIF4A1[E183Q]-EGFP-NLS
eIF4A1[R362Q]-EGFP-NLS
10 µm
B
H2B
GFP
Merge
C
Chromatin area
Time after anaphase onset [min]
eIF4A1-EGFP
eIF4A1-EGFP-NLS
eIF4A1[E183Q]-EGFP-NLS
eIF4A1[R362Q]-EGFP-NLS
D
Interphase
230.31
256.74
271.13
241.16
Nuclear area [µm²]
E
+ Rapamycin
H2B
EGFP-FRB-eIF4A1
Ki67
H2BKi67
Metaphase
Anaphase
Telophase
F
Ki67
- rapamyin
+ rapamycin
mean fluorescence intensity
G
DDX18
H2BDDX18
10µm
H
DDX18

Supplementary Figure 6: An eIF4A1-NLS version accelerates chromatin decondensation in late telophase (related to Fig. 6)

(A) Time-lapse images of H2B-mCherry HeLa cells expressing eIF4A1-EGFP-NLS, the corresponding ATPase deficient mutant eIF4A1 E183Q, or the RNA-binding deficient R362Q mutant. Time is normalized to the first anaphase frame. Scale bars: 10 µm.

(B) Images of interphase H2B-mCherry HeLa cells expressing eIF4A1-EGFP-NLS, the corresponding ATPase deficient mutant eIF4A1 E183Q, or the RNA-binding deficient R362Q mutant. Scale bars: 10 µm.

(C) Time-dependent chromatin area analysis of the experiments as in (A), including eIF4A1-EGFP, normalized to the first anaphase frame. Dots represent mean ± s.e.m. from more than 10 cells per condition.

(D) Quantitation of the nuclear area of the experiments as in (B) together with eIF4A1-EGFP. The violin plot shows the mean ± sd of more than 35 cells. Mean values are given above each transfection. Significance was tested by a two-tailed unpaired Mann-Whitney *U* test (eIF4A1-EGFP-NLS, *P=0.01; eIF4A1[E183Q]-EGFP-NLS, **P=$1.68x10^{-3}$).

(E) Ki-67 immunostaining of HeLa cells stably expressing H2B-mPlum-FKBP, transfected with EGFP-FRB-eIF4A1 without (left) or with rapamycin (right) in mitosis. Scale bars: 10 µm.

(F) Quantitation of the Ki-67 mean fluorescence intensity in metaphase, anaphase and telophase. The violin plots show the means of two independent experiments (triangles) each including at least 12 cells per condition and the overall mean ± s.e.m. Significance was tested by a two-tailed unpaired Mann-Whitney *U* test (telophase, *P=0.03).

(G) DDX18 immunostaining of HeLa cells stably expressing H2B-mPlum-FKBP, transfected with EGFP-FRB-eIF4A1 without (left) and with rapamycin (right) in mitosis. Scale bars: 10 µm.

(H) Quantitation of the DDX18 mean fluorescence intensity in metaphase, anaphase and telophase. The violin plots show the means of two independent experiments (triangles) each including at least 20 cells per condition and the overall mean ± s.e.m. Significance was tested by a two-tailed unpaired *t*-test with Welch's correction (telophase, *P=0.01).

Source data are provided as a Source Data file.

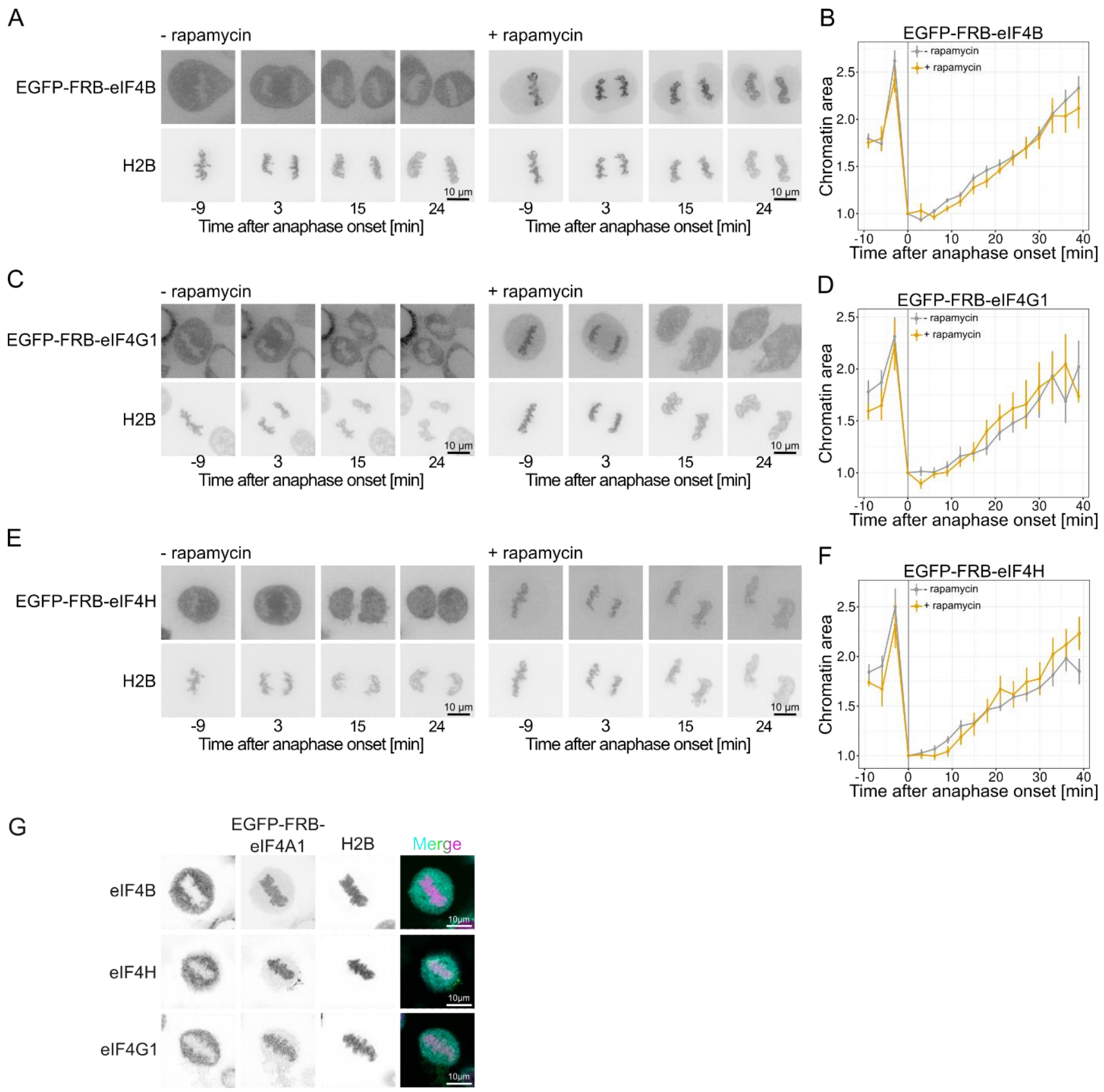

Supplementary Figure 7: Tethering other eIF4F complex members to chromosomes does not accelerate chromatin decondensation (related to Fig. 6)

(A) Time-lapse images of H2B-mPum-FKBP HeLa cells expressing EGFP-FRB-eIF4B after adding 200 nM DMSO (control, - rapamycin) or rapamycin. Time is normalized to the first anaphase frame. Scale bars: 10 µm.

(B) Time-dependent quantitation of the chromatin area of the experiments as in (A), normalized to the first anaphase frame. Dots represent mean ± s.e.m. from at least 10 cells per condition.

(C) Same as in (A) but with EGFP-FRB-eIF4G1.

(D) Same as in (B) but with EGFP-FRB-eIF4G1.

(E) Same as in (A) but with EGFP-FRB-eIF4H.

(F) Same as in (B) but with EGFP-FRB-eIF4H.

(G) eIF4B, eIF4H and eIF4G1 immunostaining (blue) of metaphase H2B-mPum-FKBP HeLa cells (chromatin in magenta) expressing EGFP-FRB-eIF4A1 (green) after adding 200 nM rapamycin. Example images are representative for 3 experiments. Scale bars: 10 µm.

Source data are provided as a Source Data file.

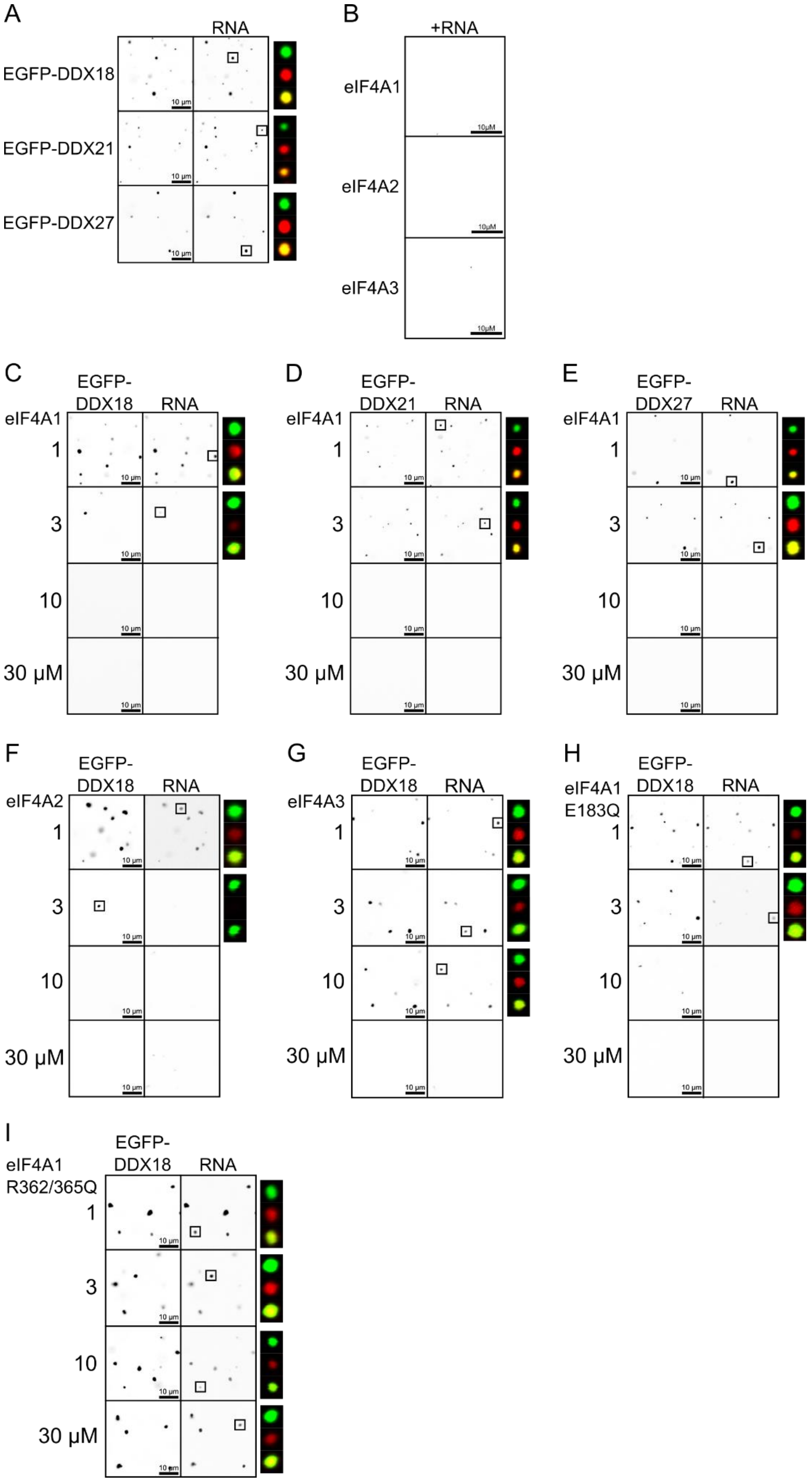

A
RNA
EGFP-DDX18
EGFP-DDX21
EGFP-DDX27
10 µm
B
+RNA
eIF4A1
eIF4A2
eIF4A3
10µM
C
EGFP-DDX18
RNA
eIF4A1
1
3
10
30 µM
D
EGFP-DDX21
RNA
eIF4A1
1
3
10
30 µM
E
EGFP-DDX27
RNA
eIF4A1
1
3
10
30 µM
F
EGFP-DDX18
RNA
eIF4A2
1
3
10
30 µM
G
EGFP-DDX18
RNA
eIF4A3
1
3
10
30 µM
H
EGFP-DDX18
RNA
eIF4A1 E183Q
1
3
10
30 µM
I
EGFP-DDX18
RNA
eIF4A1 R362/365Q
1
3
10
30 µM

Supplementary Figure 8: eIF4A1/2 destabilizes labeled RNA condensates (related to Fig. 7)

(A) *In vitro* phase separation of 10 µM recombinant EGFP-DDX18, EGFP-DDX21, or EGFP-DDX27 in the presence of 1 mg/ml RNA. For visualizing RNA, the unlabeled and Alexa-546 labeled RNA was mixed in a 9:1 molar ratio. Confocal images are representative of three independent experiments. Single RNA condensates are marked and shown as a close-up in the GFP (green), Alexa-546 (red), and merged channel (yellow as overlay of red and green). Scale bars: 10 µm.

(B) *In vitro* phase separation of 10 µM recombinant EGFP-eIF4A1, EGFP-eIF4A2, or EGFP-eIF4A3 in the presence of 1 mg/ml RNA. Confocal images are representative of three independent experiments. Scale bars: 10 µm.

(C) *In vitro* phase separation of 10 µM recombinant EGFP-DDX18 in the presence of 1 mg/ml RNA with increasing concentration of recombinant eIF4A1. For visualizing RNA, the unlabeled and Alexa-546 labeled RNA was mixed in a 9:1 molar ratio. Confocal images are representative of three independent experiments. Single RNA condensates are marked and shown as a close-up in the GFP (green), Alexa-546 (red), and merged channel (yellow as overlay of red and green). Scale bars: 10 µm.

(D) Same as in (C) but with EGFP-DDX21.

(E) Same as in (C) but with EGFP-DDX27.

(F) *In vitro* phase separation of 10 µM recombinant EGFP-DDX18 in the presence of 1 mg/ml RNA with increasing concentration of recombinant eIF4A2. For visualizing RNA, the unlabeled and Alexa-546 labeled RNA was mixed in a 9:1 molar ratio. Confocal images are representative of three independent experiments. Single RNA condensates are marked and shown as a close-up in the GFP, Alexa-546, and merged channel. Scale bars: 10 µm.

(G) Same as in (F) but with increasing concentration of recombinant eIF4A3.

(H) Same as in (F) but with increasing concentration of the recombinant ATPase deficient E183Q mutant of eIF4A1.

(I) Same as in (F) but with increasing concentration of the recombinant RNA-binding deficient R362/365Q mutant of eIF4A1.
