# Supplementary material for: The DEAD-box helicase eIF4A1/2 acts as RNA chaperone during mitotic exit enabling chromatin decondensation
Source: Nat Commun. 2025 Mar 11;16:2434. doi: 10.1038/s41467-025-57592-1 (PMC11897408; doi:10.1038/s41467-025-57592-1)
Supplement: Supplementary file 2 — Reporting Summary [file 41467_2025_57592_MOESM2_ESM.pdf]

nature portfolio

Corresponding author(s): Wolfram Antonin

Last updated by author(s): Feb 4, 2025

Reporting Summary

Nature Portfolio wishes to improve the reproducibility of the work that we publish. This form provides structure for consistency and transparency in reporting. For further information on Nature Portfolio policies, see our Editorial Policies and the Editorial Policy Checklist.

Statistics

For all statistical analyses, confirm that the following items are present in the figure legend, table legend, main text, or Methods section.

n/a	Confirmed	
☐	☒	The exact sample size (*n*) for each experimental group/condition, given as a discrete number and unit of measurement
☐	☒	A statement on whether measurements were taken from distinct samples or whether the same sample was measured repeatedly
☐	☒	The statistical test(s) used AND whether they are one- or two-sided<br>*Only common tests should be described solely by name; describe more complex techniques in the Methods section.*
☒	☐	A description of all covariates tested
☐	☒	A description of any assumptions or corrections, such as tests of normality and adjustment for multiple comparisons
☐	☒	A full description of the statistical parameters including central tendency (e.g. means) or other basic estimates (e.g. regression coefficient) AND variation (e.g. standard deviation) or associated estimates of uncertainty (e.g. confidence intervals)
☐	☒	For null hypothesis testing, the test statistic (e.g. *F*, *t*, *r*) with confidence intervals, effect sizes, degrees of freedom and *P* value noted<br>*Give P values as exact values whenever suitable.*
☒	☐	For Bayesian analysis, information on the choice of priors and Markov chain Monte Carlo settings
☒	☐	For hierarchical and complex designs, identification of the appropriate level for tests and full reporting of outcomes
☒	☐	Estimates of effect sizes (e.g. Cohen's *d*, Pearson's *r*), indicating how they were calculated

Our web collection on statistics for biologists contains articles on many of the points above.

Software and code

Policy information about availability of computer code

Data collection	Microscopy: LSM710 operated by Zen black 2.3 SP1 software; Nikon Ti2 eclipse Microscope operated by NIS-Elements AR (Version 5.20.00)<br>Western Blots: LAS4000 Image Reader (version 2.0)
Data analysis	Experiments were automatically analyzed by Fiji (version 1.54g), Python (version 3.11.1), MATLAB scripts, Cellcognition Analyser (version 1.5.2)<br>All statistical analyses were performed in R using the package ggstatsplot (version 0.12.4)

For manuscripts utilizing custom algorithms or software that are central to the research but not yet described in published literature, software must be made available to editors and reviewers. We strongly encourage code deposition in a community repository (e.g. GitHub). See the Nature Portfolio guidelines for submitting code & software for further information.

Data

Policy information about availability of data

All manuscripts must include a data availability statement. This statement should provide the following information, where applicable:

- Accession codes, unique identifiers, or web links for publicly available datasets
- A description of any restrictions on data availability
- For clinical datasets or third party data, please ensure that the statement adheres to our policy

Raw microscopy files are available from the corresponding authors upon request. Source data are provided in this article.

Research involving human participants, their data, or biological material

Policy information about studies with human participants or human data. See also policy information about sex, gender (identity/presentation), and sexual orientation and race, ethnicity and racism.

Reporting on sex and gender	*Use the terms sex (biological attribute) and gender (shaped by social and cultural circumstances) carefully in order to avoid confusing both terms. Indicate if findings apply to only one sex or gender; describe whether sex and gender were considered in study design; whether sex and/or gender was determined based on self-reporting or assigned and methods used. Provide in the source data disaggregated sex and gender data, where this information has been collected, and if consent has been obtained for sharing of individual-level data; provide overall numbers in this Reporting Summary. Please state if this information has not been collected. Report sex- and gender-based analyses where performed, justify reasons for lack of sex- and gender-based analysis.*
Reporting on race, ethnicity, or other socially relevant groupings	*Please specify the socially constructed or socially relevant categorization variable(s) used in your manuscript and explain why they were used. Please note that such variables should not be used as proxies for other socially constructed/relevant variables (for example, race or ethnicity should not be used as a proxy for socioeconomic status). Provide clear definitions of the relevant terms used, how they were provided (by the participants/respondents, the researchers, or third parties), and the method(s) used to classify people into the different categories (e.g. self-report, census or administrative data, social media data, etc.) Please provide details about how you controlled for confounding variables in your analyses.*
Population characteristics	*Describe the covariate-relevant population characteristics of the human research participants (e.g. age, genotypic information, past and current diagnosis and treatment categories). If you filled out the behavioural & social sciences study design questions and have nothing to add here, write "See above."*
Recruitment	*Describe how participants were recruited. Outline any potential self-selection bias or other biases that may be present and how these are likely to impact results.*
Ethics oversight	*Identify the organization(s) that approved the study protocol.*

Note that full information on the approval of the study protocol must also be provided in the manuscript.

Field-specific reporting

Please select the one below that is the best fit for your research. If you are not sure, read the appropriate sections before making your selection.

[x] Life sciences [ ] Behavioural & social sciences [ ] Ecological, evolutionary & environmental sciences

For a reference copy of the document with all sections, see nature.com/documents/nr-reporting-summary-flat.pdf

Life sciences study design

All studies must disclose on these points even when the disclosure is negative.

Sample size	No sample-size calculations were performed. Sample sizes were chosen as large as possible while taking into account the experimental effort required to generate the respective data. Adequate statistics has been applied throughout the manuscript in order to make sure that the observed effects are significant given the reported sample size.
Data exclusions	Pre-established exclusion criteria:<br>- no mitotic events were analysed when they showed obvisous chromosome segregation defects<br>- single frames were excluded if automated ROI definition failed<br>- for CellCognition analyses mitotic trajectories were excluded if they did not follow logic mitotic progression (e.g. annotated as dead in one frame and as metaphase in the next)
Replication	The number of replications for each experiment are provided in the figure ledgends. Experiments were performed at least twice with reproducible results.
Randomization	Not relevant as grouping was not applied.
Blinding	To minimize potential human bias, most experiments were automatically analyzed by Fiji, Python, or MATLAB scripts.

Reporting for specific materials, systems and methods

We require information from authors about some types of materials, experimental systems and methods used in many studies. Here, indicate whether each material, system or method listed is relevant to your study. If you are not sure if a list item applies to your research, read the appropriate section before selecting a response.

Materials & experimental systems

n/a	Involved in the study	
	x	Antibodies
	x	Eukaryotic cell lines
x		Palaeontology and archaeology
x		Animals and other organisms
x		Clinical data
x		Dual use research of concern
x		Plants

Methods

n/a	Involved in the study	
x		ChIP-seq
x		Flow cytometry
x		MRI-based neuroimaging

Antibodies

Antibodies used

Polyclonal Xenopus eIF4A1 antibodies were generated in rabbits using full-length Xenopus laevis eIF4A1, expressed from a pET28a vector.

commercial Antibodies: Target (company, catalog, dilution)
eIF4A1 (Cell Signaling, #2490, 1:1000)
eIF4A1/A2 (Santa Cruz, sc-377315, 1:500)
eIF4A3 (abcam, ab32485, 1:1000)
eIF4B (Cell Signaling, #13088, 1:1000)
eIF4E (Cell Signaling, #2067, 1:1000)
eIF4G1 (Cell Signaling, #2498, 1:1000)
eIF4H (Cell Signaling, #3469, 1:1000)
actin (MP Biomedicals, 691001, 1:10.000)
Mab414 antibody for detection of Nup62 (Biolegend, 902902, 1:5000)
DDX18 (bethyl laboratories, A300-636A, 1:200)
DDX27 (Santa Cruz, sc-81074, 1:200)
Ki-67 (Millipore, Mab4190, 1:100)
goat anti-rabbit IgG-HRP and goat anti-mouse IgG-HRP (Calbiochem)
donkey anti-rabbit IgG Alexa Fluor 405 (Invitrogen, #A48258)
goat anti-Mouse Alexa Fluor 488 (Invitrogen, #A-11001)
goat anti-rabbit IgG Alexa Fluor 546 (Invitrogen #A11010)
goat anti-mouse and goat anti-rabbit IgG Alexa Fluor 647 (Invitrogen, #A-21236, #A-21244)

Validation

We verified the molecular weight of the target protein by immunoblotting with extracts from Xenopus eggs or HeLa cells. Except from actin, DDX18, DDX27 and the secondary antibodies, they are also validated by knockdown or depletion experiments, which resulted in decreased signal or complete loss of the band.

eIF4A1: Fig2D
eIF4A1/A2: Fig2D
eIF4A3: Supplementary Fig. I
eIF4B: Supplementary Fig. I
eIF4E: Supplementary Fig. I
eIF4G1: Supplementary Fig. I
eIF4H: Supplementary Fig. I
actin: manufacturer webside
Mab414: https://www.biolegend.com/de-de/sean-tuckers-tests/purified-anti-nuclear-pore-complex-proteins-antibody-11498?GroupID=GROUP26
DDX18: https://www.thermofisher.com/antibody/product/DDX18-Antibody-Polyclonal/A300-636A
DDX27: https://www.scbt.com/p/ddx27-antibody-2251c2a
Ki-67: https://www.merckmillipore.com/DE/de/product/Anti-Ki-67-Antibody-clone-Ki-S5,MM_NF-MAB4190

Eukaryotic cell lines

Policy information about cell lines and Sex and Gender in Research

Cell line source(s)

Background	cell line name	Reference	PMID
HeLa Kyoto	H2B-mCherry	Moreno-Andrés et al., 2022	PMID: 32708675
HeLa Kyoto	H2B-EGFP	Llères et al. 2009	PMID: 19948497
HeLa Kyoto	H2B-mPlum-FKBP	Champion et al., 2019	PMID: 30586323
HeLa Kyoto	H2B-mPlum-FKBP, EGFP-CCDC137	generated in this study	
HeLa Kyoto	H2B-mCherry, IBB-GFP	Schmitz et al., 2010	PMID: 20711181
HeLa Kyoto	EGFP-Ki67	Cuylen et al., 2016	PMID: 27362226
RPE	hTERT-RPE1, mRFP-H2B	Dick & Gerlich 2013	PMID: 24096243

Authentication	Cells lines were obtained from the reference or generated in the lab (HeLa Kyoto H2B-mCherry and H2B-mPlum-FKBP, EGFP-CCDC137) and were not further authenticated but recognized by their morphology.
Mycoplasma contamination	All cell lines were regularly verified negative for mycoplasma contamination.
Commonly misidentified lines (See ICLAC register)	RPE and HeLa cells are not in the list of commonly misidentified cell lines.

Plants

Seed stocks	*Report on the source of all seed stocks or other plant material used. If applicable, state the seed stock centre and catalogue number. If plant specimens were collected from the field, describe the collection location, date and sampling procedures.*
Novel plant genotypes	*Describe the methods by which all novel plant genotypes were produced. This includes those generated by transgenic approaches, gene editing, chemical/radiation-based mutagenesis and hybridization. For transgenic lines, describe the transformation method, the number of independent lines analyzed and the generation upon which experiments were performed. For gene-edited lines, describe the editor used, the endogenous sequence targeted for editing, the targeting guide RNA sequence (if applicable) and how the editor was applied.*
Authentication	*Describe any authentication procedures for each seed stock used or novel genotype generated. Describe any experiments used to assess the effect of a mutation and, where applicable, how potential secondary effects (e.g. second site T-DNA insertions, mosiacism, off-target gene editing) were examined.*
