# Supplementary material for: The DEAD-box helicase eIF4A1/2 acts as RNA chaperone during mitotic exit enabling chromatin decondensation
Source: Nat Commun. 2025 Mar 11;16:2434. doi: 10.1038/s41467-025-57592-1 (PMC11897408; doi:10.1038/s41467-025-57592-1)
Supplement: Supplementary file 3 — Description of Additional Supplementary Files [file 41467_2025_57592_MOESM3_ESM.pdf]

Supplementary Data 1

Description: Supplementary Data 1 contains 4 sheets with information on the used constructs for Protein expression or cell transfections, used cell lines and siRNA oligo sequences

Supplementary Data 2

Description: This is a zipped folder containing all the custom code used for image analysis

Supplementary Data 3:

Description: This word document gives descriptions on the usage of the custom code provided in Supplementary Data 2. It also provides information on the Figures, the code was used for.
