# Supplementary material for: The DEAD-box helicase eIF4A1/2 acts as RNA chaperone during mitotic exit enabling chromatin decondensation
Source: Nat Commun. 2025 Mar 11;16:2434. doi: 10.1038/s41467-025-57592-1 (PMC11897408; doi:10.1038/s41467-025-57592-1)
Supplement: Supplementary file 6 — Supplementary Data 3 [file 41467_2025_57592_MOESM6_ESM.docx]

**Supplementary Data 3:**

**Python Code used to analyze imaging data:**

**Border_smoothness_HeLa_cluster.py**

Applied for Figure1B, E, H

This code was developed to analyze in vitro HeLa chromatin clusters in decondensation assays. Single plane images of HeLa chromatin clusters stained with DAPI, with 1 cluster per image need to be saved with the naming scheme 1.1_ WA; 1.2_ WA … 5.7_ WA etc. with the 1^st^ number describing the condition and the 2^nd^ number describing the image ID. After conversion to 8-bit tiff files, the script needs to be placed in the same folder as the files and opened in python. After definition of the number of conditions and images per condition (“condition and runs”) in the script, the code needs to be run until lane 70, loading all tiff files and generating the masks. Accurate masking can then be checked manually by looking through the masks. If a mask is not correctly calculated, this image needs to be excluded. This step is followed by execution of the second part of the code generating the plots and csv files with the measurements, with an individual sheet for each chromatin cluster. These data can be exported.

**Chromatin_area_growth_40x_objective.py**

Applied for Figure 2G and 6K

This code was developed to analyze HeLa cell trajectories in live cell imaging to analyze the chromatin area growth in widefield microscopy. For that, single cell trajectories need to be cropped from the field of view, starting at the last metaphase frame until 60 minutes after. To run the code open it in python, start the run and let it guide you through the steps. First, choose the image stack to be analyzed in the file explorer window. A window will open asking to select the chromatin channel. Select left or right and then click on “Accept”. Now, one can manually deselect the visualized ROIs in each frame, that are not accurately marking the daughter chromatin mass, using the slider and unchecking the individual boxes. Now, both daughter chromatin masses, or just one can be analyzed by confirming the saving for mass 1 and mass 2. The generated excel file will be saved where the stack is saved and provides several measurements like area or circularity. For each new trajectory analyzed, the results table will be updated with a new sheet for each daughter chromatin mass.

**Ki-67_IF_intensity.py**

Applied for Figure 4G and Supplementary Figure 4I-K

This code was developed to analyze the Ki-67 Immunofluorescence signal on HeLa mitotic chromatin in fixed samples. Therefore, squared, best in focus planes of single mitotic cells were imaged in the red and green channel (red=chromatin, green= Ki67) and converted to tiff files. When opening the code in python, a file explorer window will open to choose the tiff file to be analyzed. For the first image, a control with decent signal in mitotic exit must be chosen, to set the values for core and non-core region. A window will open asking to select the chromatin channel. Select left or right and then click on “Accept”. A window will open asking to adjust inner and outer core lengths. The inner core is shown in red and the outer in yellow. They can be adjusted moving the scale bars at the bottom. Also, the distance in pixels between the two masses of chromatin and the exact location of the centroids for its calculation are displayed. After selection of the correct sizes for the inner and outer core, the script continues by pressing “Process all images”. This will do the calculations for all the images in the same folder than the control image, using the same proportion for inner and outer core. The Window will close automatically and a new folder and excel sheet will appear in the control image file providing the result images with marked segmentation for inner-, outer-, non-core and background region. The excel file provides the data for all measurements. Manual revision of the annotated regions allows deleting data of erroneous assignations.

all scripts run in Python (version 3.11.1)

**ImageJ macros used to analyze imaging data:**

**gfp_mitosis_actionbar.ijm**

Applied for Figure 6 B,D,F,H,J and Supplementary Figure 5, 6C, 7B,D,F

This ImageJ macro was developed to analyze single, high resolution mitotic tracks with a stable H2B-mCherry or H2B-mPlum and a transient eGFP signal fused to a target protein. For this macro, the ImageJ plugin “Action Bar” needs to be installed (<https://imagej.net/media/plugins/action-bar/mutterer_workshop.pdf>). When installed as described, the script can be opened through the Action Bar plugin which then generates buttons on the ImageJ window. Beginning with “Open File”, one chooses the input file, a two-channel time stack, via the file explorer. Next, with the “Split and crop” button, one chooses a suitable position for the generated rectangle to capture the target cell through mitosis. Confirming with “OK” the macro splits the channel and generates the crops. With “Define mitotic frames” one is asked to define a destination directory to save the data that will be generated in the following steps. Next, one fills in the stack number from last metaphase to chromatin decondensation. With pressing “OK”, the stacks will be cropped and saved for each channel. Now there is an additional option to “Invert LUT” and save these stacks as well. The “Quantify” button sets an auto threshold in the chromatin channel to identify the chromatin ROI in each frame. The individual ROIs added to the ROI manager then need manual revision and every falsely annotated ROI can be deleted individually. To continue “OK” needs to be pressed. Now, a ROI needs to be positioned in the cytoplasm that stays cytoplasmic through mitosis, before pressing “OK”. Finally, the automated mitotic phase assignment can be checked in the results table, which is also saved at the defined output directory and provides the measured values for the chromatin mass and the GFP signal.

**h2b_mitosis.ijm**

Applied for Figure 2F and Supplementary Figure 2

This ImageJ macro was developed to analyze single, high resolution mitotic tracks with a stable H2B-mCherry signal to investigate chromatin decondensation. It needs to be opened in ImageJ which automatically opens it in the Notebook. Next, the cell trajectory file with a single channel chromatin signal needs to be opened. Starting the code, it first asks to define a destination directory to save the data that will be generated in the following steps. Next, one chooses a suitable position for the generated rectangle to capture the target cell through mitosis. Confirming with “OK” the macro splits the channel and generates the crops. Next, one fills in the stack number from last metaphase to chromatin decondensation. With pressing “OK”, the stacks will be cropped and saved. This code also automatically saves an inverted stack for which the contrast can be manually adjusted before pressing “OK”. Now, an auto threshold is set in the chromatin channel to identify the chromatin ROI in each frame. The individual ROIs added to the ROI manager then need manual revision and every falsely annotated ROI can be deleted individually. To continue “OK” needs to be pressed. Finally, the automated mitotic phase assignment can be checked in the results table, which is also saved at the defined output directory and provides the measured values for the chromatin mass.

**DDX18_27_IF_intensity.ijm**

Applied for Figure 3 B,D,H and Supplementary Figure 6 F,H

This code was developed to analyze the DDX18, DDX27 Immunofluorescence and RNAclick signal on HeLa mitotic chromatin in fixed samples. It needs to be opened in ImageJ which automatically opens it in the Notebook. First, all individual mitotic cells need to be cropped and saved as tiff in a separate folder with individual naming scheme including treatment. Starting the script, one chooses the tiff files folder by using the browse button and chooses a name for the results folder which will be generated. Now, the script will loop through every image, pausing for the user to confirm the generated ROI(s) in the chromatin channel. Falsely annotated ROIs can be deleted in the ROI manager. This script will only analyze one daughter chromatin mass, if the cell has already gone through anaphase onset. The ROI chosen to be analyzed is by default the first ROI of the ROI manager, hence chosen by chance. Next, a small circular ROI needs to be set in the cytoplasm of the green channel. There, it is important to choose a region with background signal and no bright spots in it. By pressing “OK” the script will automatically save the results and open the next image. Once all images are analyzed, the script will loop through all saved csv files and generate one resulting file with all measurements including the individual file names of the cells to be able to trace back the measurements to the image.

All scripts run in Fiji (version 1.54g)
